# Investigating the Molecular Mechanism of H3B-8800: A Splicing Modulator Inducing Preferential Lethality in Spliceosome-Mutant Cancers

**DOI:** 10.3390/ijms222011222

**Published:** 2021-10-18

**Authors:** Angelo Spinello, Jure Borišek, Luca Malcovati, Alessandra Magistrato

**Affiliations:** 1 National Research Council of Italy, Institute of Materials Foundry (CNR-IOM) c/o SISSA, Via Bonomea 265, 34136 Trieste, Italy; aspinello@sissa.it; 2 National Institute of Chemistry, Theory Department, Hajdrihova 19, 1000 Ljubljana, Slovenia; jure.borisek@ki.si; 3 Department of Hematology, IRCCS S. Matteo Hospital Foundation, 27100 Pavia, Italy; luca.malcovati@unipv.it; 4 Department of Molecular Medicine, University of Pavia, 27100 Pavia, Italy

**Keywords:** splicing modulators, H3B-8800, spliceosome-mutant cancer, leukemia, molecular dynamics

## Abstract

The SF3B1 protein, part of the SF3b complex, recognizes the intron branch point sequence of precursor messenger RNA (pre-mRNA), thus contributing to splicing fidelity. SF3B1 is frequently mutated in cancer and is the target of distinct families of splicing modulators (SMs). Among these, H3B-8800 is of particular interest, as it induces preferential lethality in cancer cells bearing the frequent and highly pathogenic K700E SF3B1 mutation. Despite the potential of H3B-8800 to treat myeloid leukemia and other cancer types hallmarked by SF3B1 mutations, the molecular mechanism underlying its preferential lethality towards spliceosome-mutant cancer cells remains elusive. Here, microsecond-long all-atom simulations addressed the binding/dissociation mechanism of H3B-8800 to wild type and K700E SF3B1-containing SF3b (^K700E^SB3b) complexes at the atomic level, unlocking that the K700E mutation little affects the thermodynamics and kinetic traits of H3B-8800 binding. This supports the hypothesis that the selectivity of H3B-8800 towards mutant cancer cells is unrelated to its preferential targeting of ^K700E^SB3b. Nevertheless, this set of simulations discloses that the K700E mutation and H3B-8800 binding affect the overall SF3b internal motion, which in turn may influence the way SF3b interacts with other spliceosome components. Finally, we unveil the existence of a putative druggable SF3b pocket in the vicinity of K700E that could be harnessed in future rational drug-discovery efforts to specifically target mutant SF3b.

## 1. Introduction

Pre-messenger RNA (pre-mRNA) splicing, i.e., the removal of the intronic sequences from a nascent pre-mRNA transcript and the ligation of exons to generate a mature mRNA, is orchestrated by the spliceosome (SPL), a multi-megadalton protein/RNA machinery [1]. The SPL preserves genomic integrity by precisely recognizing/selecting specific sequences such as the 5′ and the 3′ splice sites (5′SS and 3′SS, respectively), at the exons/introns boundaries, and the branch point sequence (BPS), which contains the conserved branch point adenosine (BPA, the nucleophile of the first splicing step) [2,3,4,5].

Splicing dysregulation is a hallmark of a wide variety of human diseases [6], including cancer and neurodegeneration. Recurrent somatic mutations of splicing factors (SFs) are implicated in splicing deregulation and are linked to hematologic malignancies (i.e., myelodysplastic syndromes (MDS) [7], chronic myelomonocytic leukemia (CMML) [8], and chronic lymphocytic leukemia (CCL) [9]), and some solid tumors (e.g., breast cancer and uveal melanoma [10]). These heterozygous mutations affect several SFs, including SF3B1 (the most frequently mutated), U2AF1, SRSF2, and ZRSR2, which are involved in the selection of critical pre-mRNA sequences to ensure splicing fidelity [11]. These mutants affect splicing, resulting in aberrant pre-mRNA transcripts that can: (i) be degraded via nonsense-mediated mRNA decay, leading to a downregulation of gene and protein expression; or (ii) be translated into protein isoforms and/or defective proteins. Although the frequent cancer-associated mutations of SFs trigger aberrant splicing through apparently different mechanisms, they often decrease the production of canonical mRNA suitable to be translated into functional proteins. As a result, mutated SFs provoke an altered genetic background commonly referred as a status of “spliceosome sickness” [12].

The SF3b complex, a central component of the U2 small nuclear RNP (snRNP), plays a key role in the recognition of the BPA. Mutations of the SF3B1 protein, the main SF3b component, alter BPS recognition fidelity, triggering the selection of cryptic 3′SS, and finally leading to aberrant splicing [12]. The most recurrent K700E SF3B1 mutation often occurs in patients affected by myelodysplastic syndromes, such as myelodysplasia with ring sideroblasts [13]. In this scenario, the SPL is emerging as an appealing target for cancer treatment via small molecules, also due to the increased understanding of its mechanism provided by recent structural [14,15,16], functional [17,18], and computational studies [19,20]. SF3b was the first SF for which structural information in complex with small-molecules splicing modulators (SMs) were solved [14,15,16]. These SMs were initially discovered from natural products and later derivatized in drug discovery studies. The best-characterized SMs belong to the spliceostatin, pladienolide, and herboxidiene families, all sharing a tripartite organization with a common central diene motif [21]. Although these structurally-related SMs target the BPA recognition site, laying at the interface of the SF3B1 and PHFA5 proteins, they differently affect splicing regulation [17] and SF3b functional dynamics [20], suggesting their splicing modulation may result from a more subtle inhibition mechanism than a pure competition with the pre-mRNA [22].

Among the SMs targeting SF3b is H3B-8800 (called hereafter H3B), recently evaluated in a phase I clinical trial. H3B affects both canonical and aberrant splicing in cancer cells bearing SF3B1 mutations in in vitro assays [23]. H3B has also shown a preferential antitumor activity in cell cultures and patient-derived xenograft models presenting cancer-associated mutations of SF3B1 and SRSF2 [23]. In addition, the recently concluded phase I clinical trial demonstrated that this drug candidate has an acceptable adverse effect profile and identified a subset of patients who could benefit from its treatment [24].

In spite of its potential therapeutic benefit for patients affected by hematologic malignancies, the mechanism underlying H3B’s preferential lethality towards mutant cancer cells bearing the K700E-SF3B1 (^K700E^SF3B1) mutation remains unclear at the molecular level. It is appealing to suppose that H3B achieves selectivity by having a greater affinity for mutant SF3B1, but it is unclear how this could occur considering that the site of the K700E mutation (and other common cancer-associated SF3B1 mutations) is located far from the SM binding site. In addition, competition binding assays of H3B with E7107 (a structurally related SMs targeting SF3b) were similar to SF3b complexes containing wild type (WT) or ^K700E^SF3B1 (hereafter referred as ^WT^SF3b and ^K700E^SF3b, respectively) [23], suggesting that the K700E mutation does not affect H3B’s binding affinity to ^WT^SF3b and ^K700E^SF3b. In this study, we explored this question by unlocking the H3B binding/dissociation mechanism to ^WT^SF3b and ^K700E^SF3b via all-atom molecular dynamics (MD) and enhanced sampling simulations. Our results reveal that H3B binds with similar kinetic and thermodynamic properties to WT and mutant SF3b, thus providing additional support to the proposal that its preferential lethality leverages a general vulnerability of cancer cells containing mutant SFs. Nevertheless, we also disclosed that the K700E mutation and/or H3B binding affect the overall internal motion of the complex, which may result in altered interactions/regulation of other SPL components. Finally, we pinpoint the presence of an allosteric pocket nearby the K700E mutation site, which may be harnessed in future drug-discovery studies aimed at devising novel selective inhibition strategies tackling mutant SF3b.

## 2. Results

**Figure 1:**
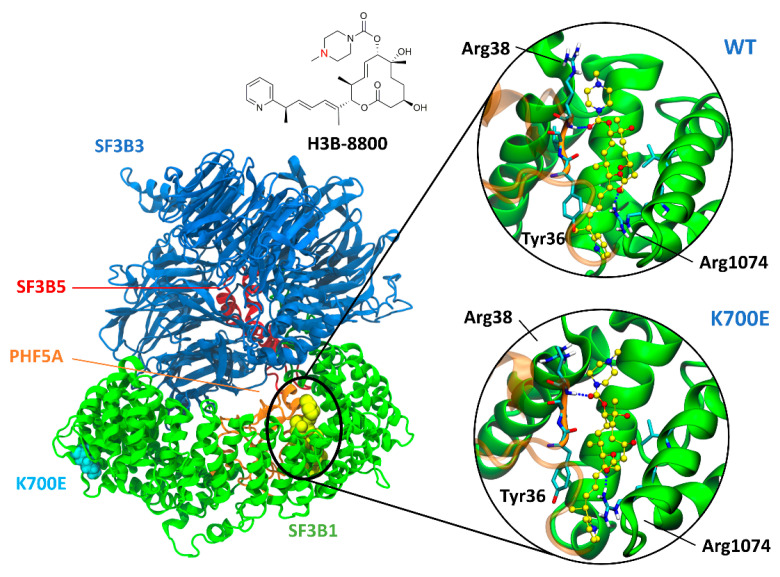
Representative structure of SF3b [25] in complex with the splicing modulator H3B-8800, as obtained from a cluster analysis of molecular dynamics (MD) simulations trajectories. SF3B1 (green), SF3B3 (blue), SF3B5 (red), and PHF5A (orange) are shown as cartoons, and the K700E and modulators sites in light blue and yellow van der Waals spheres. Insets show H3B-8800, highlighted in ball and sticks with carbon atoms depicted in yellow, in complex with wild type (WT) and K700E SF3B1. Sketch of H3B-8800 with the nitrogen atom subject to possible protonation marked in red (top).

No crystal structure of H3B in complex with the SPL is currently available, therefore the model of H3B bound with SF3b was built by superposing the H3B’s macrolide moiety to that of E7107, a structurally related SM for which a cryo-EM structure in complex with SF3b is available [14]. To exhaustively explore the source of H3B’s preferential lethality in cancer cells possessing a mutant SF3B1, we have built a model of H3B bound to ^WT^SF3b and ^K700E^SF3b (hereafter named as ^WT^SF3b_H3B_ and ^K700E^SF3b_H3B_, respectively). Both systems underwent 1 μs-long, explicitly solvated MD simulations reaching structural convergence after 200 ns (Figure S1). The MD trajectories revealed that H3B possesses similar binding poses and binding free energies (ΔG_b_) in both ^WT^SF3b_H3B_ and ^K700E^SF3b_H3B_ (Figure 1 and Table 1).

### 2.1. Structural, Dynamical, and Energetic Characterization of H3B Binding to SF3b

The diene moiety, the key pharmacophore trait shared by all SMs targeting SF3B1, contributes to stabilizing the drug at the SF3B1/PHF5A interface by establishing π-stacking interactions with Tyr36@PHF5A and Arg1074@SF3B1. Furthermore, H3B persistently hydrogen (H)-bonds with the backbone of Arg38@PHF5A and the side chain of Arg1074@SF3B1 (Figure 1, Table S1). A per-residue decomposition analysis of the ΔG_b_ (Table 1 and Table S2) enables us to dissect the residues more strongly contributing to H3B binding [14,15]. Remarkably, this analysis assigns a key role to Arg1074@SF3B1, whose guanidinium group markedly contributes to the ΔG_b_ by engaging a persistent H-bond with the H3B carbonyl moiety, and cation-π interaction with its diene moiety. These results are in line with the larger decrease in the H3B antiproliferative activity toward cancer cells carrying Arg1074His as compared to Tyr36Cys mutation [23]. Overall, since H3B occupies the same BPA binding pocket and assumes a very similar binding pose to E7107 [14,15], is likely to stall SF3B1 in an “open” conformation as suggested for other SMs [15]. This could in turn impair the recruitment of the BPS intronic sequence.

At physiological pH, the neutral and positively-charged state of H3B are predicted to be equally likely (see Methods Section 5.1) [27]. As such, H3B in its cationic state can be protonated on the methylated nitrogen atom of the piperazine ring (Figure 1). Since different drugs’ protonation states can strongly affect their molecular recognition by biological targets [28,29], we also performed 1 μs-long MD simulations for the positively charged H3B (H3B^+^) in complex with either ^WT^ or ^K700E^SF3b (called hereafter ^WT^SF3b_H3B+_ and ^K700E^SF3b_H3B+_). As a result, H3B^+^ has a similar binding pose (RMSD of 1.2 and 0.6 Å for the WT and the K700E mutant, respectively, Figure S2), resulting in only a slightly higher ΔG_b_ to ^K700E^SF3b_H3B+_ as compared to ^WT^SF3b_H3B+_ (Table S3).

### 2.2. H3B Dissociation Mechanism and Kinetics Properties

**Figure 2:**
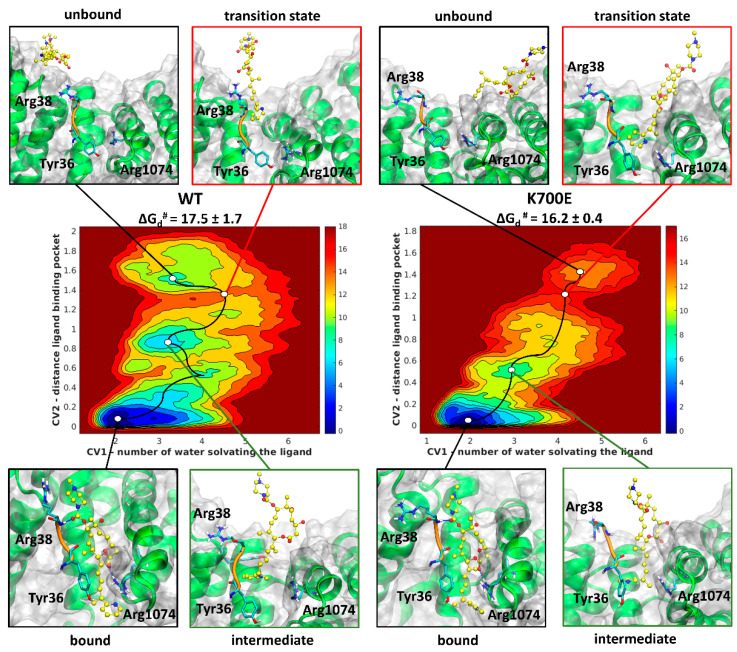
Free energy surface (FES, kcal/mol) and free energy barrier (ΔG_d_^#^) for the dissociation of H3B from SF3b-containing ^WT^ and ^K700E^SF3B1 (^WT^SF3b and ^K700E^SF3b, respectively) as obtained from metadynamics simulations. The FES, ranging from dark blue to dark red with contour lines every 1.5 kcal/mol, is plotted as a function of two collective variables (CV): CV1 number of water molecules solvating the ligand, and CV2 distance between the centers of mass of the ligand and the binding pocket. The minimum free energy path is shown with a black line, with minima marked by a white circle. Insets show the structures of relevant bound, intermediate, transition, and unbound states encountered along the dissociation path. SF3B1 (green) and PHF5A backbone connecting Tyr36 to Arg38 (orange) are shown as new cartoons, while H3B is depicted in ball and stick with carbon atoms shown in yellow.

In order to obtain insights on the residence time of H3B in the target site [30,31], which differences may be related to the distinct activity of this SM in mutant cancer cells, we have also inspected its binding/dissociation kinetics to/from ^WT^SF3b and ^K700E^SF3b, by performing metadynamics (MTD) simulations. MTD allows the acceleration of the ligand dissociation from SF3b within the time frame of an all-atom MD simulation, while exploring the mechanism along with the associated free energy landscape. Remarkably, even the dissociation of H3B from ^WT^SB3b or ^K700E^SB3b is characterized by a very similar mechanism and free energy barriers (ΔG_d_^#^s) (17.5 ± 1.7 vs. 16.2 ± 0.4 kcal/mol_,_ for ^WT^SB3b or ^K700E^SB3b, respectively, Figure 2). To the best of our knowledge, no kinetic data are available on SF3b inhibitors. Nevertheless, order-of-addition experiments suggested that these SMs are characterized by very slow dissociation kinetics [32].

A detailed analysis of the residues contributing to stabilizing the transition state structures reveals that the breakage of the hydrophobic and electrostatic interactions between H3B, Tyr36-Arg38@PHF5A, and Arg1074@SF3B1, contribute to the increase of ΔG_d_^#^ (Table S4). Conversely, its interactions with Arg38@PHF5A, Arg1075, Val1078, and Asn1079@SF3B1, stabilize the transition state for its dissociation from ^WT^SF3b_H3B_ and ^K700E^SF3b_H3B_, respectively. Besides the small differences observed in the transition states, H3B follows a similar dissociation path in both WT and mutant systems, visiting alike intermediate states where its diene and pyridine moieties engage hydrophobic and stacking interactions with the SF3B1 and PHF5A residues (e.g., Val1078@SF3B1 and Pro39@PHF5A), while the macrolide is fully solvated (Figure 2 and Table S4).

### 2.3. SF3b Protein Dynamics

The C-terminal domain of SF3B1, the main component of the SF3b complex, is composed of a sequence of 20 HEAT repeats domains [25]. This gives SF3B1 a highly flexible nature, which becomes very sensitive to perturbation induced by mutations and the binding of different SMs as revealed by previous MD simulations [19,20]. Hence, in this study, we have performed a principal component analysis (PCA, Figure S3) to investigate how the binding of H3B affects the large-scale collective motions of ^WT^SF3b and ^K700E^SF3b. As a result, no significant differences were observed on the SF3B1/PHF5A proteins, for apo (undrugged) SF3b models (^WT^SF3b_apo_ and ^K700E^SF3b_apo_), where a similar spring-like motion of SF3B1 occurs [19]. Conversely, upon H3B binding small differences in ^WT^SF3b_H3B_ and ^K700E^SF3b_H3B_ appear, resulting in an enhanced motion of the central portion of the SF3B1 ring in the mutant system (Figure S3). In particular, the general counterclockwise motion, which is the prevalent movement in all systems investigated, is perturbed in ^K700E^SF3b_H3B_, where the C- and N-terminations concertedly move toward the central part of the SF3B1 ring.

**Figure 3:**
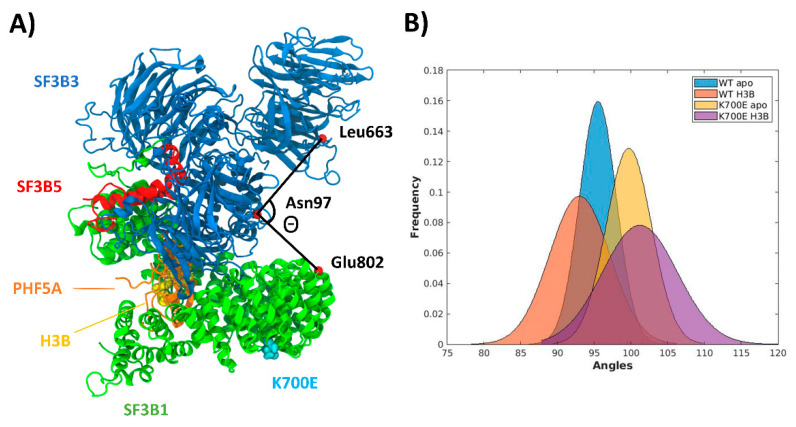
(**A**) Bending angle (Θ), defined by the Cα atoms of Leu663 and Asn97 in SF3B3 and Glu802 in SF3B1. SF3B1 (green), SF3B3 (blue), SF3B5 (red), and PHF5A (orange) are shown as cartoons while the K700E mutation site is shown as cyan van der Waals spheres. (**B**) Distribution of Θ angle (deg) values sampled during molecular dynamics simulations trajectories for the wild type apo SF3b (^WT^SF3b_apo,_ light blue), for WT SF3b in complex with H3B (^WT^SF3b_H3B_, orange), apo SF3b containing ^K700E^SF3B1 (^K700E^SF3b_apo_, yellow) and SF3b containing ^K700E^SF3B in complex with H3B (^K700E^SF3b_H3B_, purple).

Moreover, when considering the collective motions of the entire SF3b complex we observed a similar bending of the SF3B3 BPB domain with respect to the central portion of the SF3B1 ring (Figure S4). Nevertheless, by monitoring the distribution of the bending angle, Θ (Figure 3), we noticed that the K700E mutation triggers a shift of Θ distributions at larger values, as compared to the ^WT^SF3b models. In addition, H3B binding induces an opposite shift and makes the distributions broader, with the widest angles and the broadest distribution occurring in the presence of both H3B and K700E. In this scenario, it is tempting to suggest that H3B not only affects the motion of SF3b regions distant from its binding pockets, but it may also influence the SF3b internal dynamics and/or in turn the function (inhibition/activation) of the nearby interacting proteins assembled within the SPL complex.

### 2.4. Detection of Druggable Allosteric Pockets

Aiming to explore novel and selective inhibition strategies for SF3B1 mutations recurrent in hematologic malignancies we employed a cavity detector algorithm to pinpoint druggable (allosteric) sites, distinct from the BPA binding cavity. Targeting these allosteric pockets with small molecules may alter the SF3B1 conformational remodeling, occurring upon BPA recruitment, thus allosterically inhibiting its selection.

The search for a druggable site was performed by inspecting the structure of the most representative cluster of SF3B1 and PHF5A proteins extracted from ^WT^SF3b_apo_ and ^K700E^SF3b_apo_ MD trajectories [33,34,35]. Remarkably, three putative binding pockets located in a similar position for both SF3b isoforms were identified (Figure S5): the most druggable allosteric pocket (druggability scores are listed in Table S5), Site1, laid between the HEAT-repeats H3-H4 of SF3B1 near the K700E mutation site (H6); Site2, placed between the H1–H3 and PHF5A, and Site 3, between H16–H17, near the SMs binding pocket (Figure S5 and Table S5). Interestingly, Site 1, located nearby the K700E residue, may be exploited to identify a ^K700E^SF3B1-specific allosteric drug.

## 3. Discussion

Recently, all-atom simulations have contributed to unravel several facets of the splicing mechanism [36,37,38]. Following and complementing previous studies, here we exploited biased and unbiased MD simulations to comprehensively characterize the binding/dissociation mechanism of H3B, an SM currently in clinical trials, which preferentially triggers lethality in cancer cells and patient-derived xenograft models bearing mutations of the splicing factors SF3B1 and SRSF2 [23].

Since no crystal structure of the H3B modulator in complex with SF3b has been solved to date and aiming to unlock the source of H3B preferential lethality towards cancer cells, we characterized its binding pose and dissected the residues that contribute most to the binding towards ^WT^SF3b and ^K700E^SF3b by performing extensive µs-long MD simulations. Albeit H3B similarly binds to ^WT^SF3b and ^K700E^SF3b from both structural and thermodynamic points of view, some of the residues that contribute to stabilizing the drug (e.g., Tyr36@PHF5A, Arg1074, and Val1078@SF3B1) were pinpointed as critical for H3B activity in mutational studies [18,39], confirming the reliability of our findings. Furthermore, the dissociation mechanism and kinetic properties (i.e., the residence times) for H3B unbinding from ^WT^SF3b and ^K700E^SF3b were also alike (H3B possesses dissociation free energy barriers of about 17 kcal/mol in both systems).

Previous MD simulations have highlighted that the functional dynamics of the SF3b complex, and in particular the flexible SF3B1 protein, is influenced by the presence of distinct SMs or carcinogenic mutations [19,20]. Thus, we also inspected whether internal SF3b motion could be affected by H3B binding and/or the presence of the K700E mutation. Indeed, by monitoring the bending between the BPB domain of SF3B3 and the SF3B1 protein, we observed that they modify the SF3b internal dynamics triggering an average shift in the Θ angle distribution (Figure 3) of 4.7° in the presence of the mutation, and of 8.2° when both the mutation and the drug are present. Since the SPL is a huge and dynamic molecular machinery composed of hundreds of proteins and snRNAs involved in a constant remodeling, we inspected the proteins in close contact with the SF3b complex in SPL structures deposited in the Protein Data Bank (PDB). As a result, we observed that the BPB domain of SF3B3 interacts with the Ski2-like helicase (Brr2) in the early B^act^ complex (pdb ID 5Z58) [40] (Figure S6). This position may also be accessible to other proteins in subsequent not yet structurally resolved structures of the SPL cycle. As such, it is tempting to suggest that the K700E mutation and/or H3B binding may differently modulate SF3b interactions with Brr2 and/or other proteins interacting with it along the SPL cycle.

Thus, the possibility of selectively modulating cancer-associated splicing defects by targeting mutant SFs remains an appealing opportunity. Along this line, we identified a druggable allosteric pocket nearby the K700E mutation in SF3B1 (Figure S5), which may be exploited in future structure-based drug discovery studies aimed at devising novel inhibitory strategies to selectively target SF3b containing the most frequent mutations in patients affected by hematologic malignancies.

## 4. Conclusions

In the present study, all-atom simulations unambiguously demonstrated at the atomistic level that the preferential lethality of the H3B modulator towards cancer cells bearing mutated SFs does not build on its different affinity or longer residence time to ^K700E^SB3b as compared to ^WT^SF3b. These data further support the proposition suggesting that H3B selectivity relies on its opportunistic ability to synergize and increase the splicing related-burden provoked by SF3B1 (and other SFs) mutations, which trigger spliceosome-mutant cancer cells death. Being SFs mutations heterozygous, spliceosome-mutant cancer cells exclusively rely on functional splicing as operated by WT SPL for survival, and any intervention that further reduces it (the effect of H3B summed to that of the SF3B1 mutant) [41], makes cancer cells containing mutant SFs more susceptible to small-molecule modulation. Owing to the overwhelming implication of aberrant splicing in cancer onset, the possibility of selectively targeting spliceosome-mutant cancers, based on a rational understanding of the structural and dynamical alterations provoked to each splicing factor by somatic mutations, remains a challenging yet appealing pharmacological opportunity.

## 5. Materials and Methods

### 5.1. Model Building

We have built six models starting from an equilibrated structure from a previous work of SF3b in complex with the SM E7107 [20], a structurally related SM for which a cryo-EM structure is available (PDB ID: 5ZYA) [14]. In this model, we considered all components of the structure deposited in the PDB database (id 5ZYA), which includes the proteins SF3B1, SF3B3, SF3B5, PHF5A, and 3 Zn^2+^ ions. The model of H3B in complex with SF3b (^WT^SF3b_H3B_) was built by superposing the diene and macrolide moiety of this SM to that of E7107 trapped in the structure. By removing this modulator from the structure, we have obtained the apo structure (^WT^SF3b_apo_). The protonation state of H3B was evaluated using the Epik software [27]. At physiological pH, the neutral and positively charged state of H3B, protonated on the methylated nitrogen of the piperazine ring, are predicted as equally likely. Thus, we have also built a model including the protonated H3B modulator (^WT^SF3b_H3B+_). Finally, we have modified the three WT models (^WT^SF3b_apo_, ^WT^SF3b_H3B_, ^WT^SF3b_H3B+_) in their corresponding mutated system (^K700E^SF3b_apo_, ^K700E^SF3b_H3B_, ^K700E^SF3b_H3B+_) by changing Lys700 into Glu700 in the SF3B1 protein. On the resulting six models, we have performed µs-long molecular dynamics simulations.

### 5.2. Molecular Dynamics Simulations

Molecular Dynamics (MD) simulations were performed with Gromacs2020.2 [42]. The AMBER-ff14SB force field (FF) [43] was employed for proteins and the general Amber FF (GAFF) for the H3B modulator [44]. The Zn^2+^ ions were modeled with the cationic dummy atoms approach developed by Pang [45] as in our previous simulations [19,20,36,38]. The partial charges of H3B were calculated by performing a Hartree-Fock optimization using a 6-31G* basis set with Gaussian09 [46]. RESP charges were then obtained with the Antechamber [47] module included AmberTools18 [48]. The six investigated models were embedded in a 10 Å layer of TIP3P water molecules [49], 3 Zn^2+^, and 43 Na^+^ counterions counting up to 320,000 atoms. The topologies were built with AmberTools18 and were subsequently converted in a GROMACS format using the software acpype [50].

All the MD simulations underwent a slow equilibration protocol, described previously [20]. In particular, our models were minimized using the steepest descent algorithm. Then, the systems were smoothly annealed from 0 to 300 K with a temperature gradient of 50 K every 2 ns and for a total of 12 ns using a Berendsen barostat [51]. In this phase, only water molecules and Na^+^ ions were allowed to move, while the rest was subjected to harmonic position restraints with a force constant of 1000 kJ/mol nm^2^. MD simulations were performed in the isothermal-isobaric NPT ensemble, at a temperature of 300 K, under the control of a velocity-rescaling thermostat [52]. Then, the barostat was switched to Parrinello-Rahman [53] and the position restraints were restricted only to the backbone of the protein’s atoms. These were gradually decreased in three consecutive runs of 10 ns each, during which the force constant was set to 1000, 250, 50 kJ/mol nm^2^, respectively. Finally, all the restraints were released, and the production runs were performed for 1 µs for each of the models, reaching overall cumulative 6 μs of MD. Productive MD simulations were performed in the NPT ensemble. A LINCS algorithm [54] was used to constrain the bonds involving hydrogen atoms, while the particle mesh Ewald method [55] was used to account for long-range electrostatic interactions with a cutoff of 10 Å. An integration time step of 2 fs was used in all simulations.

### 5.3. Metadynamics Simulations

In order to investigate the dissociation kinetics of the H3B modulator, we have performed FF-based metadynamics (MTD) simulations. In particular, MTD runs of 50–100 ns were performed using GROMACS 2020.2 [42] and the PLUMED 2.0 plugin [56]. Two collective variables (CVs) were employed: the first (CV1) describes the solvation of the modulator and is defined by counting the number of water oxygen atoms that are comprised within 3 Å of H3B center of mass. The second (CV2) corresponds to the distance between the center of masses (COM) of the heavy atoms composing the modulator binding pocket and H3B.

Gaussian hills, having a height of 1 kJ/mol and widths of 0.10 and 0.02, were added, respectively, for CV1 and CV2 every 5 ps of MTD. In order to restrain the exploration of the free energy surface a harmonic wall at 30 Angstrom was used for CV2. For each MTD simulation, we performed three replicas, starting from different initial velocities and frames, as extracted from the MD trajectories. The uncertainty in the corresponding dissociation free energy barriers (ΔG_d_^#^) were estimated taking the standard deviation of each barrier obtained out of the three replicas, as in previous simulations MTD studies [57,58].

### 5.4. Molecular Mechanics-Generalized Surface Area (MM-GBSA)

Binding free energies (ΔG_b_) between SF3b and H3B modulator, along with a per-residue decomposition analysis, were calculated using the molecular mechanics-generalized born surface area (MM-GBSA) method [26] using MMPBSA.py program [59]. Errors are reported as their corresponding standard error of mean. A salt concentration of 0.1 M was used and the igb value was set to 8, as successfully employed in other studies [60,61]. MM-GBSA calculations were performed on 100 frames. Finally, the conformational entropic contribution to the ΔG_b_ was not taken into account, as it was reported that it does not improve the quality of the results [62].

### 5.5. Analysis

MD trajectories were visualized and analyzed with the VMD software [63]. Root-mean-square deviation (RMSD) was performed with tools implemented in GROMACS 2020.2 [42]. All analyses were performed on the equilibrated part of the trajectories (200–1000 ns). Moreover, we looked for possible binding pockets able to host potential allosteric SMs, following an established computational protocol [34,35] using the SiteMap software [64]. This algorithm identifies potential binding pockets by linking together distinct ‘site points’ that contribute to protein–ligand binding. SiteMap then characterizes binding sites through a combination of chemico-physical and solvation properties that are calculated for each site point. Then, site points are aggregated into larger pockets when they are in close proximity and may be bridged in solvent-exposed regions by the ligand. The drug-binding and druggability properties of the identified pockets are evaluated using Site and Druggability Scores. A Site Score of at least 0.80 is able to accurately discriminate among drug-binding and non-drug-binding sites. The Druggability Score, which takes more into account the hydrophilic/hydrophobic nature of the binding pocket, is able to distinguish challenging and undruggable targets from druggable ones, the latter characterized by higher values. The search of druggable pockets was carried out on the representative structures extracted from our MD simulations using cluster analysis.

### 5.6. Principal Component Analysis

Principal component analysis (PCA) was performed using the cpptraj module of Ambertools 18 [48] in order to extract the “essential dynamics” of the investigated models. Briefly, PCA is able to capture the large-scale collective motions of biomolecules in MD simulations [65]. These key motions have been identified by calculating the mass-weighted covariance matrix of the Cα atoms. The resulting eigenvectors are characterized by the largest eigenvalues accounting for the direction of the most important motions (principal components, PCs) sampled during our MD simulation. The Normal Mode Wizard plugin present in the VMD software was used to visualize the essential dynamics along the principal eigenvectors and to show the arrows highlighting their corresponding directions.

## Figures and Tables

**Table 1:** Binding free energies (ΔG_b,_ kcal/mol) of H3B-8800 to SF3b complex containing wild type (^WT^SF3b_H3B_) and K700E SF3B1 (^K700E^SF3b_H3B_) and their per-residue decomposition (kcal/mol) calculated with the Molecular Mechanics Generalized Surface Area (MM-GBSA) method [26]. Residues involved in the stabilization of the H3B-8800 binding pose are marked in yellow, in light green, and in dark green when their contribution to ΔG_b_ is smaller than −1.0 kcal mol, when it ranges between −1.0 and −2.0 kcal/mol, and when it is larger than −2.0 kcal/mol, respectively. Standard errors of mean are smaller than 0.1 kcal/mol.

^WT^SF3b_H3B_ ΔG_b_ −45.3 ± 0.4			^K700E^SF3b_H3B_ ΔG_b_ −44.9 ± 0.3		
Residue	ΔG_b_ Side Chain	ΔG_b_ total	Residue	ΔG_b_ Side Chain	ΔG_b_ Total
Lys1067	−0.5	−1.1	Lys1067	−0.3	−1.1
Lys1071	−0.6	−1.8	Lys1071	−0.4	−1.1
Arg1074	−4.4	−4.4	Arg1074	−4.6	−4.8
Arg1075	−1.4	−1.3	Arg1075	−0.9	−1.7
Val1078	−2.0	−2.0	Val1078	−1.9	−1.9
Val1110	−0.5	−0.5	Val1110	−0.9	−1.0
Val1114	−1.2	−1.3	Val1114	−1.0	−1.1
Tyr1157	−2.0	−1.6	Tyr1157	−1.7	−1.0
Tyr36	−2.2	−1.9	Tyr36	−2.5	−2.0
Val37	−1.2	−2.1	Val37	−1.5	−2.5
Arg38	−1.2	−2.1	Arg38	−0.6	−1.7

## Data Availability

The data presented in this study are available on request from the corresponding author.

## Supplementary Materials

The following are available online at https://www.mdpi.com/article/10.3390/ijms222011222/s1.
