# Supplementary material for: Investigating the Molecular Mechanism of H3B-8800: A Splicing Modulator Inducing Preferential Lethality in Spliceosome-Mutant Cancers
Source: Int J Mol Sci. 2021 Oct 18;22(20):11222. doi: 10.3390/ijms222011222 (PMC8540225; doi:10.3390/ijms222011222)
Supplement: Supplementary file 1 [file ijms-22-11222-s001.zip › ijms-1383090-supplementary.pdf]

Supporting Information

Investigating the molecular mechanism of H3B-8800: a splicing modulator inducing preferential lethality in spliceosome-mutant cancers

Angelo Spinello [1], Jure Borisek [2], Luca Malcovati [3] and Alessandra Magistrato [1,*]

1) National Research Council of Italy, Institute of Materials (CNR-IOM) c/o SISSA, via Bonomea 265, 34136, Trieste, Italy

2) National Institute of Chemistry, Hajdrihova 19, 1000, Ljubljana, Slovenia

3) Department of Molecular Medicine, University of Pavia & Unit of Precision Hematology Oncology, IRCCS S. Matteo Hospital Foundation, Pavia, Italy

*Correspondence: alessandra.magistrato@sissa.it

Figure S1 to S6 Pag. 2-7

Table S1 to S5 Pag. 8-13

References Pag. 14

Supplementary Figures

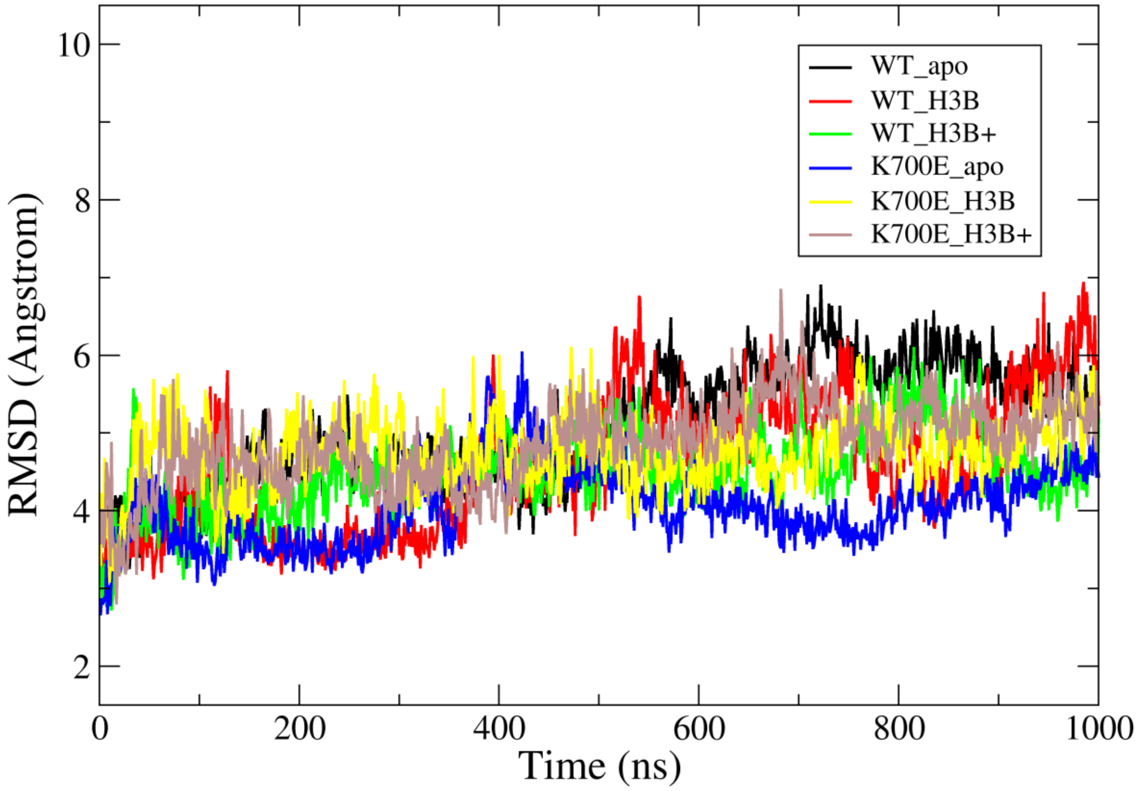

Figure S1. Root mean square deviation (RMSD, Å) vs simulation time (ns) calculated on the wild type (WT, black) apo SF3b ($^{WT}SF3b_{apo}$), WT SF3b in complex with neutral H3B-8800 (H3B, $^{WT}SF3b_{H3B}$, red) and positively charged H3B (H3B+, $^{WT}SF3b_{H3B+}$) (green), apo SF3b containing K700E SF3B1 ($^{K700E}SF3b_{apo}$, blue), SF3b, containing K700E SF3B1, in complex with neutral H3B ($^{K700E}SF3b_{H3B,}$ yellow) and H3B+ ($^{K700E}SF3b_{H3B+,}$ tan).

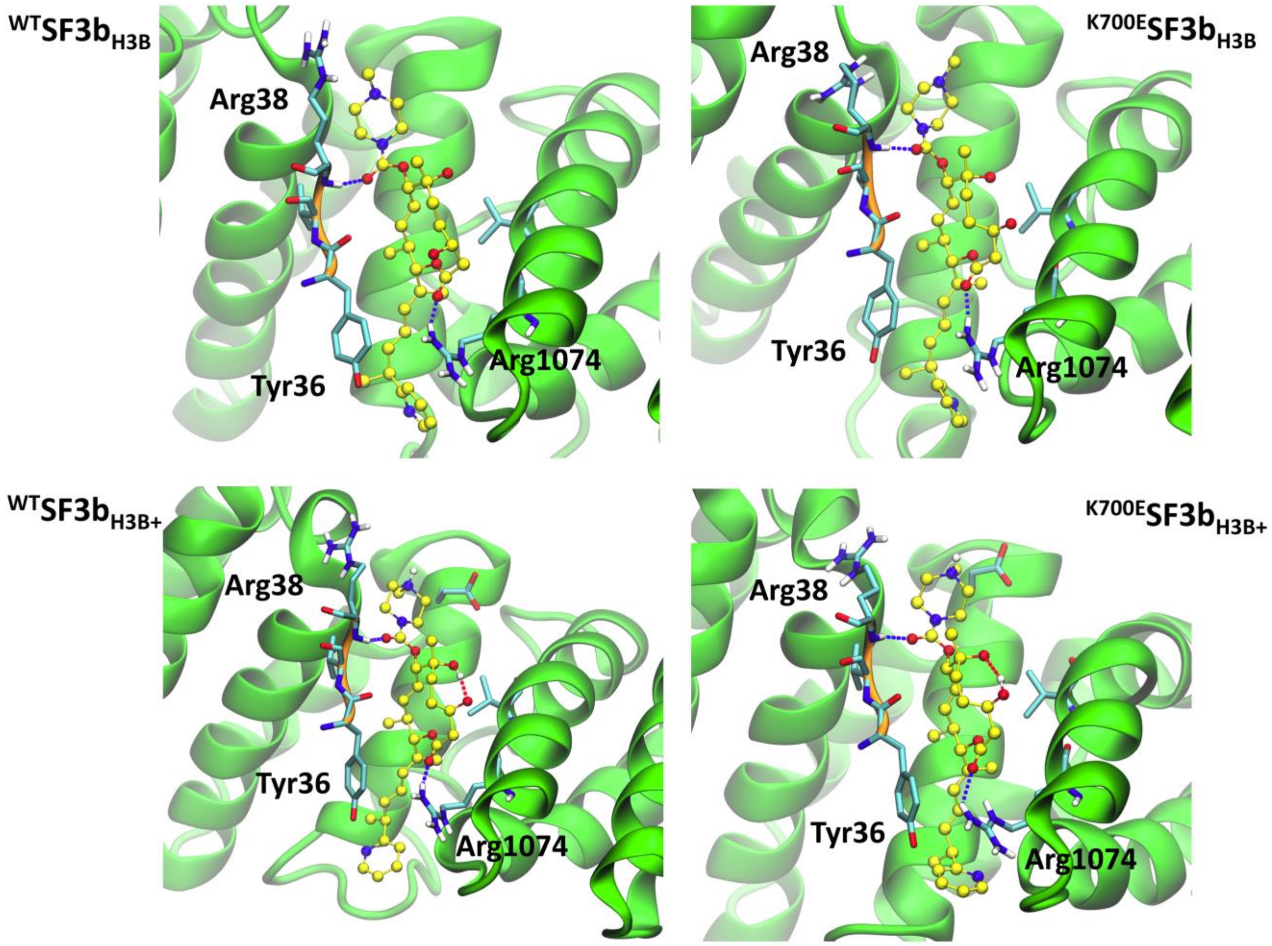

Figure S2. Representative structure of neutral and protonated H3B-8800 (H3B and H3B+, respectively) in complex with the WT and K700E SF3B1-containing SF3b ($^{WT}SF3b_{H3B+}$ and $^{K700E}SF3b_{H3B+}$, respectively) as obtained from a cluster analysis of molecular dynamics simulation trajectories. SF3B1 (green), and PHF5A (orange) are shown as cartoons. Residues important residues for H3B binding are shown in licorice. The drug is shown in ball and sticks with yellow carbon atoms.

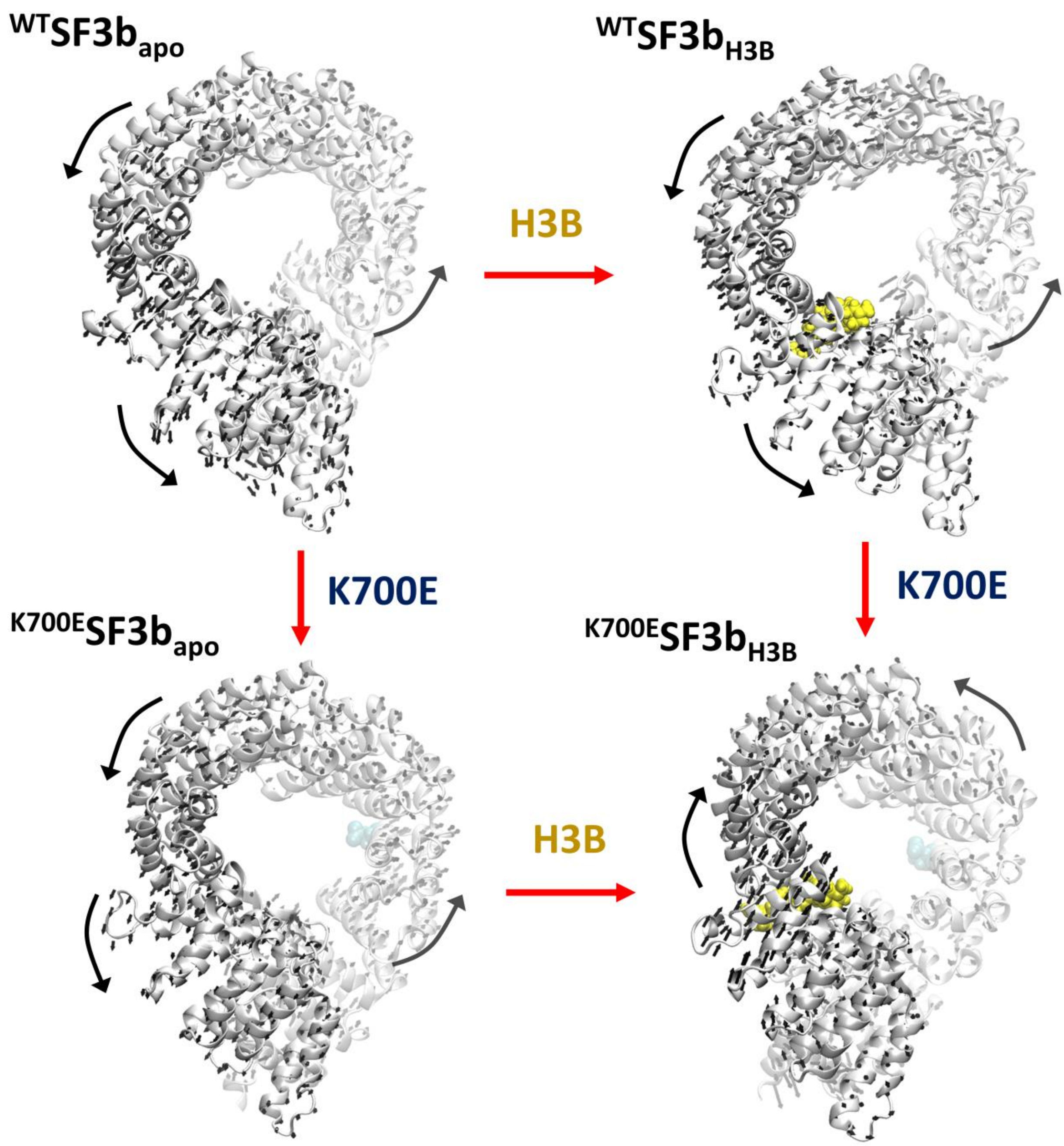

Figure S3. Essential dynamics of SF3B1 as revealed by principal component analysis (PCA) extracted from the analysis of the molecular dynamics simulation trajectory. The first principal component (PC1) of the wild type (WT) apo SF3b model ($^{WT}SF3b_{apo}$), the wild type SF3b model in complex with H3B-8800 drug (H3B, $^{WT}SF3b_{H3B}$), the K700E SF3B1-containing apo SF3b ($^{K700E}SF3b_{apo}$) and K700E SF3B1-containing SF3b bound to H3B ($^{K700E}SF3b_{H3B}$) models are reported. The SF3B1 protein is shown in white new cartoon with gray arrows indicating the direction and the amplitude of the Cα atoms motion along PC1. The K700E mutation site and H3B are shown in cyan and yellow van der Waals spheres, respectively. Black arrows highlight the overall SF3B1 movement.

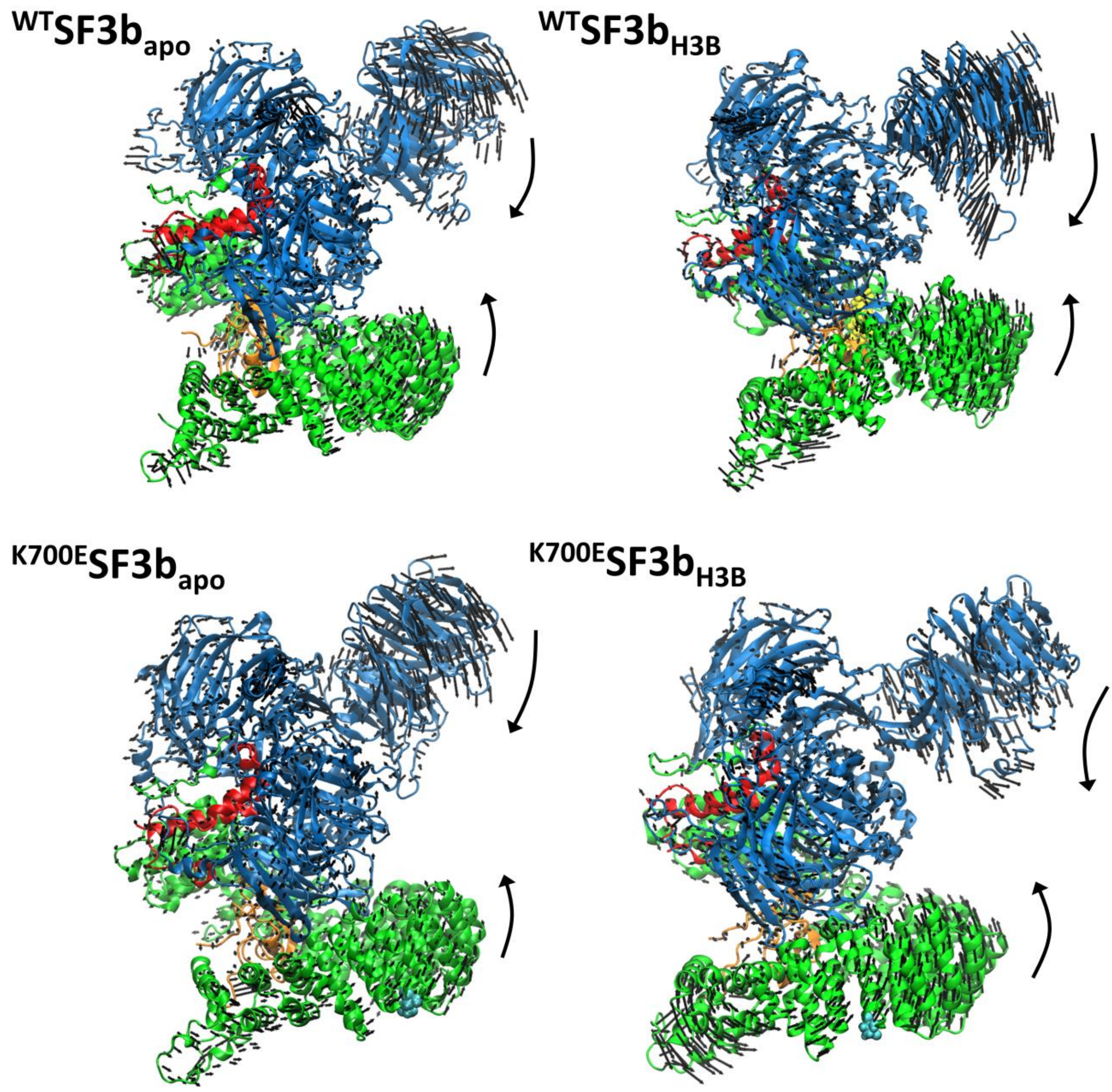

Figure S4. Essential dynamics of the SF3b complex as revealed by principal component analysis (PCA) of the molecular dynamics simulation trajectories. The first principal component (PC1) is shown for the wild type apo SF3b model (^{WT}SF3b$_{apo}$), the wild type SF3b model in complex with H3B-8800 drug (H3B, ^{WT}SF3b$_{H3B}$), in the K700E SF3B1-containing apo SF3b (^{K700E}SF3b$_{apo}$) and K700E SF3B1-containing SF3b bound to H3B (^{K700E}SF3b$_{H3B}$). SF3B1 (green), SF3B3 (blue), SF3B5 (red) and PHF5A (orange) are shown as cartoons and the K700E mutation site and H3B are shown as light blue and yellow van der Waals spheres, respectively. Small black arrows are used to highlight the motion of Cα atoms along PC1. Large black arrows highlight the overall SF3b movement.

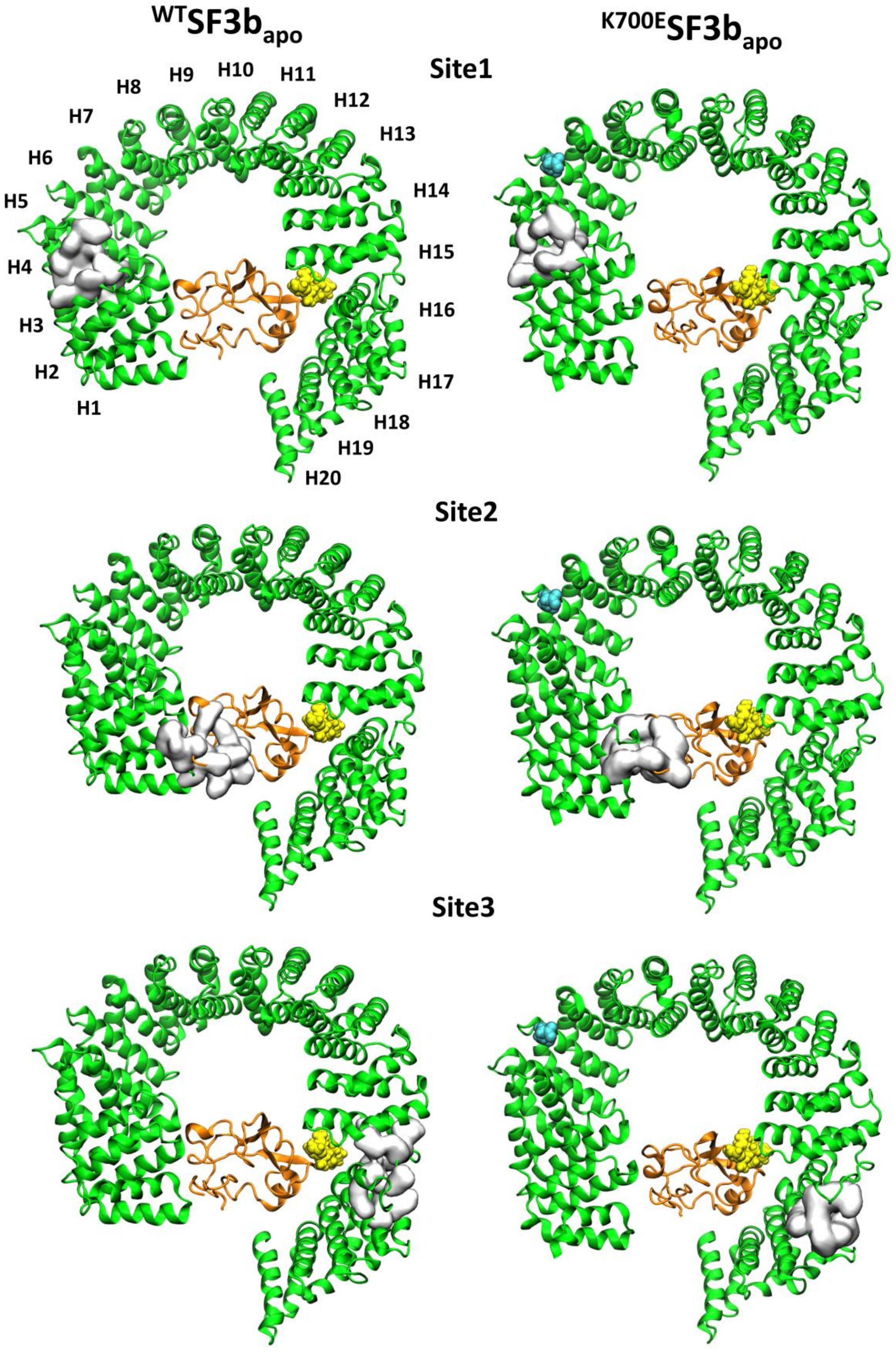

Figure S5. The positions of the three most druggable allosteric binding pockets obtained with the SiteMap programs [1] by analyzing representative clusters obtained from the molecular dynamics trajectories: the pockets are shown as a white surface. SF3B1 and PHF5A are shown as green and orange new cartoons, respectively. the K700E mutation and H3B binding site are shown as cyan and yellow van der Waals spheres, respectively.

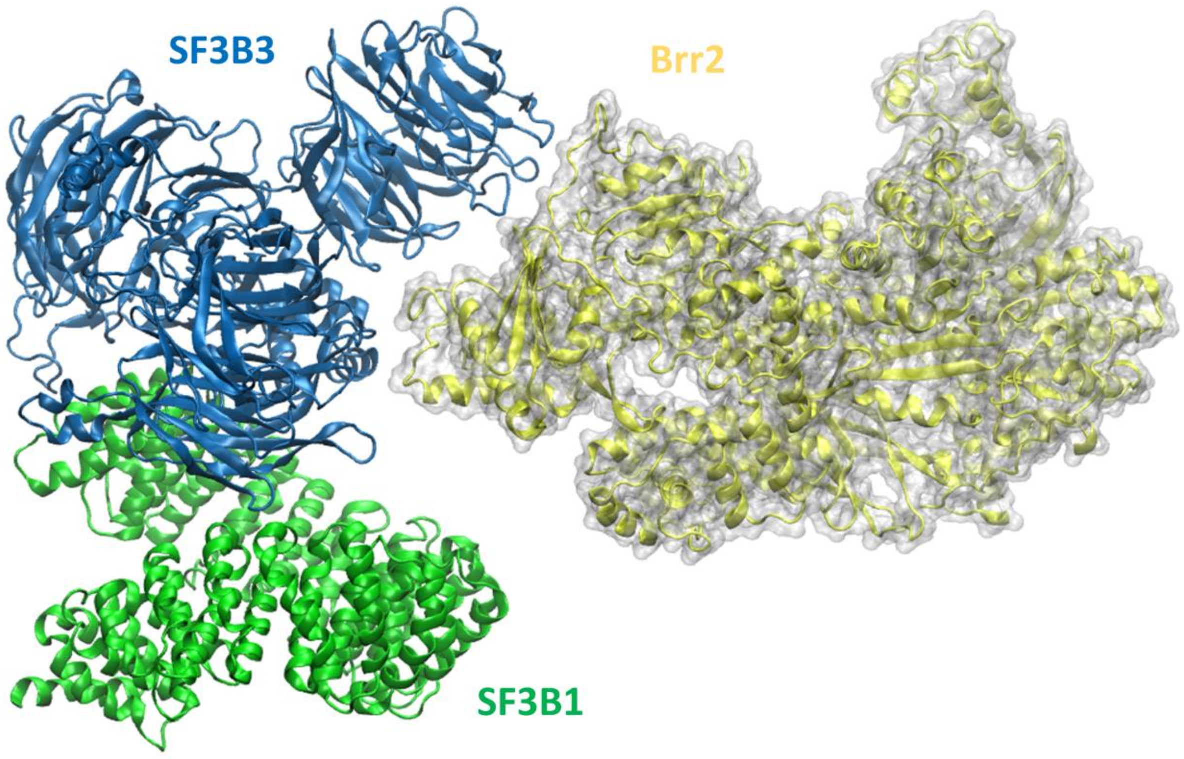

Figure S6. Structure of SF3B3 (blue cartoons), SF3B1 (green cartoons) and Ski2-like helicase (Brr2, yellow cartoons and surface) as extracted by an early Bact complex (PDB code 5Z58) [2], showing Brr2 in close proximity with the BPB domain of SF3B3.

Supplementary Tables

Table S1. PHF5A and SF3B1 protein residues involved into the formation of persistent hydrogen bonds (H-bonds) with H3B-8800 drug as observed in molecular dynamics simulations trajectories of wild type SF3b in complex with H3B-8800 drug (H3B, $^{WT}SF3b_{H3B}$) and K700E SF3B1-containing SF3b bound to H3B ($^{K700E}SF3b_{H3B}$). The persistence (%) and the average length (Å) of the H-bonds are also reported.

^{WT}**SF3b$_{H3B}$**	**Persistence (%)**	**Average distance (Å)**
Arg38@H ^{PHF5A} - H3B@O	64.9	2.86
Arg1074@HH ^{SF3B1} - H3B@O	63.2	2.82
^{K700E}**SF3b$_{H3B}$**	**Persistence (%)**	**Average distance (Å)**
Arg38@H ^{PHF5A} - H3B@O	76.2	2.85
Arg1074@HH ^{SF3B1} - H3B@O	59.2	2.84
^{WT}**SF3b$_{H3B+}$**	**Persistence (%)**	**Average distance (Å)**
Arg38@H ^{PHF5A} - H3B@O	58.6	2.87
Arg1074@HH ^{SF3B1} - H3B@O	58.4	2.82
^{K700E}**SF3b$_{H3B+}$**	**Persistence (%)**	**Average distance (Å)**
Arg38@H ^{PHF5A} - H3B@O	66.7	2.87
Arg1074@HH ^{SF3B1} - H3B@O	45.8	2.86

Table S2. Molecular Mechanics Generalized Surface Area (MM-GBSA) binding free energies (ΔG_b) along with their per-residue decomposition free energy (kcal/mol) and its van der Waals (vdW) and the electrostatic contribution (El) [3] for the wild type SF3b model in complex with H3B-8800 drug (H3B, $^{WT}SF3b_{H3B}$), and the K700E SF3B1-containing SF3b bound to H3B ($^{K700E}SF3b_{H3B}$). The residues contributing to ΔG_b by more than 1.0 kcal/mol are listed. Residues involved in the stabilization of the binding pose are marked in light green if their contribution ranges between -1.0 and -2.0 kcal/mol and in dark green if their contribution is smaller than -2.0 kcal/mol. Standard errors of mean, if not reported, are below 0.1 kcal/mol.

**$^{WT}SF3b_{H3B}$ $\Delta G_b = -45.3 \pm 0.4$**			
**Residue**	**ΔG_b total**	**vdW**	**El**
Lys1067	-1.1	-1.3	-1.4
Lys1071	-1.8	-1.2	-2.2 ± 0.14
Arg1074	-4.4	-3.7	-8.7 ± 0.17
Arg1075	-1.3	-1.4	0.1
Val1078	-2.0	-1.8	-0.1
Val1114	-1.3	-1.0	-0.1
Tyr1157	-1.6	-3.6	-0.5
Tyr36	-1.9	-3.4	-0.9
Val37	-2.1	-1.6	-1.7
Arg38	-2.1	-1.9	-1.9 ± 0.11
**$^{K700E}SF3b_{H3B}$ $\Delta G_b = -44.9 \pm 0.3$**			
**Residue**	**ΔG_b total**	**vdW**	**El**
Lys1067	-1.1	-1.1	-1.7
Lys1071	-1.1	-1.1	-2.1 ± 0.11
Arg1074	-4.8	-4.1	-8.5 ± 0.15
Arg1075	-1.7	-1.1	-2.5
Val1078	-1.9	-1.7	-0.2
Val1110	-1.0	-0.8	-0.2
Val1114	-1.1	-0.9	-0.1
Tyr1157	-1.0	-3.0	-0.7
Tyr36	-2.0	-3.9	-0.6
Val37	-2.5	-1.9	-1.6
Arg38	-1.7	-1.3	-1.4

Table S3. Molecular Mechanics Generalized Surface Area (MM-GBSA) binding free energies (ΔG_b) per-residue decomposition (kcal/mol) [3] of the entire residue and the side chain are reported. Only the residues contributing to ΔG_b by more than 1.0 kcal/mol are listed. This analysis is done for the wild type SF3b model in complex with H3B-8800 (H3B, $^{WT}SF3b_{H3B}$), and for the K700E SF3B1-containing SF3b bound to H3B ($^{K700E}SF3b_{H3B}$). For comparison, we also show the same residue in the related systems containing positively charged H3B (H3B+) ($^{WT}SF3b_{H3B}$/$^{WT}SF3b_{H3B+}$ and $^{K700E}SF3b_{H3B}$/$^{K700E}SF3b_{H3B+}$). Residues involved in the stabilization of the binding pose are marked in yellow if their contribution to the ΔG_b is larger than -1.0 kcal mol, in light green if it is between -1.0 and -2.0 kcal/mol and in dark green if it is smaller than -2.0 kcal/mol. Standard errors of mean are below 0.1 kcal/mol.

**$^{WT}SF3b_{H3B}$ ΔG_b -45.3 ± 0.4**			**$^{WT}SF3b_{H3B+}$ ΔG_b -43.5 ± 0.4**		
**Residue**	**ΔG_b side chain**	**ΔG_b total**	**Residue**	**ΔG_b side chain**	**ΔG_b total**
Lys1067	-0.5	-1.1	Lys1067	-0.4	-0.9
Lys1071	-0.6	-1.8	Lys1071	-0.3	-0.8
Arg1074	-4.4	-4.4	Arg1074	-4.4	-4.7
Arg1075	-1.4	-1.3	Arg1075	-0.9	-1.7
Val1078	-2.0	-2.0	Val1078	-1.9	-1.9
Val1114	-1.2	-1.3	Val1114	-0.9	-1.2
Tyr1157	-2.0	-1.6	Tyr1157	-2.8	-3.0
Tyr36	-2.2	-1.9	Tyr36	-2.3	-2.0
Val37	-1.2	-2.1	Val37	-1.6	-2.1
Arg38	-1.2	-2.1	Arg38	-0.7	-1.2

**$^{K700E}SF3b_{H3B}$ ΔG_b -44.9 ± 0.3**			**$^{K700E}SF3b_{H3B+}$ ΔG_b -46.9 ± 0.5**		
**Residue**	**ΔG_b side chain**	**ΔG_b total**	**Residue**	**ΔG_b side chain**	**ΔG_b total**
Lys1067	-0.3	-1.1	Lys1067	-0.5	-1.4
Lys1071	-0.4	-1.1	Lys1071	-0.2	-0.9
Arg1074	-4.6	-4.8	Arg1074	4.2	-4.5
Arg1075	-0.9	-1.7	Arg1075	-1.1	-1.8
Val1078	-1.9	-1.9	Val1078	-1.9	-1.9
Val1110	-0.9	-1.0	Val1110	-0.6	-0.8
Val1114	-1.0	-1.1	Val1114	-1.4	-1.6
Tyr1157	-1.7	-1.0	Tyr1157	-2.8	-3.5
Ile1158	-0.1	-0.2	Ile1158	-0.6	-1.0
Tyr36	-2.5	-2.0	Tyr36	-0.6	-1.7
Val37	-1.5	-2.5	Val37	-1.2	-1.6
Arg38	-0.6	-1.7	Arg38	-1.2	-1.4

Table S4. Molecular Mechanics Generalized Surface Area (MM-GBSA) binding free energies (ΔG_b) per-residue decomposition (kcal/mol) [3], of the entire residue and the side chain for the bound and transition states, as extracted from metadynamics simulations. This analysis is done for the wild type SF3b model in complex with H3B-8800 drug (H3B, $^{WT}SF3b_{H3B}$), and the K700E SF3B1-containing SF3b bound to H3B ($^{K700E}SF3b_{H3B}$). Residues involved in the stabilization of the binding pose are marked in yellow if their contribution to the ΔG_b is larger than -1.0 kcal mol, in light green if it is between -1.0 and -2.0 kcal/mol and in dark green if it is smaller than -2.0 kcal/mol. Standard errors of mean are below 0.1 kcal/mol.

	**$^{WT}SF3b_{H3B}$ bound state ΔG_b -40.1 ± 0.8**		**$^{WT}SF3b_{H3B}$ INT ΔG_b -20.5 ± 0.4**		**$^{WT}SF3b_{H3B}$ TS ΔG_b -12.8**	
**Residue**	**ΔG_b side chain**	**ΔG_b total**	**ΔG_b side chain**	**ΔG_b total**	**ΔG_b side chain**	**ΔG_b total**
**SB3B1**						
Lys1067	-0.3	0.7	-	-	-	-
Lys1071	-0.4	-1.3	-0.1	0.1	-	-
Arg1074	-3.4	-3.5	-0.2	-0.2	-	-
Arg1075	-1.1	-1.0	-0.3	-0.4	-	-
Val1078	-1.4	-1.3	-1.4	-1.4	-0.8	-0.7
Val1110	-0.5	-0.5	-	-	-	-
Val1114	-1.1	-1.2	-0.6	-0.7	-	-
Phe1153	-0.4	-0.5	-0.5	-0.5	-	-
Tyr1157	-1.6	-1.6	-1.1	-1.2	-0.6	-0.7
**PHF5A**						
Tyr36	-2.2	-1.8	-1.0	-1.1	-	-
Val37	-1.4	-2.2	-0.4	-0.1	-0.1	-0.2
Arg38	-1.0	-1.9	-1.5	-0.8	-1.6	-1.5
Pro39	-0.6	-0.9	-0.7	-1.0	-0.1	-0.2

	^{K700E}SF3b$_{H3B}$ bound state ΔG_b -42.2 ± 0.4		^{K700E}SF3b$_{H3B}$ INT ΔG_b -23.6 ± 0.3		^{K700E}SF3b$_{H3B}$ TS ΔG_b -16.6	
**Residue**	**ΔG_b side chain**	**ΔG_b total**	**ΔG_b side chain**	**ΔG_b total**	**ΔG_b side chain**	**ΔG_b total**
**SB3B1**						
Lys1067	-0.4	-1.2	0	-0.1	0	-0.1
Lys1071	-0.5	-1.7	0.1	-0.1	0.2	-0.6
Arg1074	-4.5	-4.5	-0.4	-0.7	0.1	-0.3
Arg1075	-1.2	-1.0	-0.8	-1.4	-1.3	-1.6
Val1078	-2.0	-1.9	-2.0	-2.2	-1.6	-2.3
Asn1079	0	-0.1	0.7	0.9	-1.4	-1.7
Val1110	-0.8	-0.9	-0.4	-0.5	-	-
Val1114	-1.2	-1.2	-1.4	-1.5	-0.7	-0.7
Phe1153	-0.3	-0.4	-0.5	-0.6	-0.2	-0.2
Tyr1157	-1.6	-0.9	-0.8	-0.7	-0.3	-0.3
**PHF5A**						
Tyr36	-2.1	-1.8	-1.6	-0.8	-1.1	-0.3
Val37	-1.3	-2.1	-0.9	-0.4	-0.4	-0.2
Arg38	-0.9	-1.9	-0.6	0	-0.1	-0.1
Pro39	-0.4	-0.6	-1.6	-1.5	-0.2	-0.2

Table S5. List of residues forming the three putative allosteric pockets for the SF3b complex containing the wild type (WT) ^{WT}SF3B1 and ^{K700E}SF3B1 protein, along with their Site and Druggability Score values. A Site Score of at least 0.80 is able to accurately discriminate among drug-binding and non-drug-binding sites. The Druggability Score, which takes more into account the hydrophilic/hydrophobic nature of the pocket, is able to distinguish challenging and undruggable targets from druggable ones, the latter characterized by higher values (see Methods section for more details) [1].

^{WT}SF3b$_{apo}$		Site Score	Druggability Score
**Site1**	**SF3B1**<br>His572, Leu575, Val576, Glu579, Leu582, Ile597, Met613, Asp616, Tyr623, Val624, Thr627, Thr628, Arg630, Ala631	1.06	1.07
**Site2**	**SF3B1**<br>Phe466, Lys505, Asn506, Gly507, Pro509, Glu548<br>**PHF5A**<br>Ile9, Phe10, Arg12, Val31, Tyr54, Ile60, Asp83, Pro86, Lys87, Ile88, Val89, Asn90, Ser93	0.98	1.00
**Site3**	**SF3B1**<br>Leu1063,Phe1081, Ile1088, Val1093, Leu1094, Leu1098, Arg1109, Thr1112, Ala1115, Ile1116, Val1119, Ala1131, Leu1132, Val1147	0.95	0.96
**^{K700E}SF3b$_{apo}$**		**Site Score**	**Druggability Score**
**Site1**	**SF3B1**<br>His572, Leu575, Val576, Glu579, Thr612, Met613, Asp616, Asn619, Tyr623, Val624, Thr627, Arg630, Ala631	1.00	0.99
**Site2**	**SF3B1**<br>Lys468, Lys505, Asn506, Arg512, Glu545, Gln547, Glu548, His550, Leu551<br>**PHF5A**<br>Lys13, Glu48, Tyr51, Tyr54, Val89, Asn90, Gly92, Ser93, Ser94	0.97	0.98
**Site3**	**SF3B1**<br>Thr1096, Asn1099, Asn1100, Arg1106, Arg1109, Thr1113, Leu1132, Glu1135, Asn1142, Val1143, Lys1149, Ser1150	0.93	0.74

References

1. Halgren, T. A., Identifying and characterizing binding sites and assessing druggability. *J. Chem. Inf. Model.* **2009,** 49, (2), 377-89.
2. Zhang, X.; Yan, C.; Zhan, X.; Li, L.; Lei, J.; Shi, Y., Structure of the human activated spliceosome in three conformational states. *Cell Res.* **2018,** 28, (3), 307-322.
3. Kollman, P. A.; Massova, I.; Reyes, C.; Kuhn, B.; Huo, S.; Chong, L.; Lee, M.; Lee, T.; Duan, Y.; Wang, W.; Donini, O.; Cieplak, P.; Srinivasan, J.; Case, D. A.; Cheatham, T. E., 3rd, Calculating structures and free energies of complex molecules: combining molecular mechanics and continuum models. *Acc. Chem. Res.* **2000,** 33, (12), 889-97.
